# Development of a Milli-Kelvin Thermostatic Bath for High-Precision Temperature Sensor Calibration

**DOI:** 10.3390/s26165210

**Published:** 2026-08-17

**Authors:** Yupei Zhang, Yuting Xiong, Jing Cheng, Dongxu Wu, Jian Sun, Yating Lu, Jinlong Zhou, Chengke Wang, Jie Shan, Bin Zhou

**Affiliations:** 1 Zhejiang Key Laboratory of Acoustic Intelligent Sensing and Advanced Measurement, Zhejiang Institute of Quality Sciences, Hangzhou 310018, China; beibei210363@126.com (Y.Z.);; 2 Key Laboratory of In-Situ Measurement of Ministry of Education, China Jiliang University, Hangzhou 310018, China

**Keywords:** thermostatic bath, temperature calibration, milli-kelvin, NTC, temperature control

## Abstract

For most temperature sensors, measurement traceability must be established via a comparison method within a thermostatic environment. To address the limitations of domestic constant temperature baths in meeting the calibration requirements for high-precision temperature sensors in fields such as semiconductors, a thermostatic bath device with milli-kelvin (mK) level temperature control capability has been developed. First, a novel system scheme was designed to create a sub-cooled condition using a refrigeration machine, while temperature balance was maintained by adjusting the heating power. Second, a high-precision temperature measurement module was designed using a thermistor sensor and a resistance ratio measurement method; measurement accuracy was improved by employing a bidirectional constant current source. Finally, an optimized temperature control logic was proposed to achieve rapid temperature regulation. Experimental results demonstrate that the device operates effectively within a (5–50) °C range, with stability better than 0.003 °C/h and uniformity superior to 0.003 °C. The device has been calibrated by a professional organization and meets the verification requirements for high-precision temperature sensors.

## 1. Introduction

The precise measurement and stable control of temperature serve as a cornerstone for a vast array of scientific research and modern industrial technologies. As one of the seven base quantities in the International System of Units, the accurate dissemination and reproduction of thermodynamic temperature values are fundamental to the reliability and advancement of fields ranging from fundamental physics experiments to high-end precision manufacturing [1].

Beyond specific performance indicators, thermal stability has been widely recognized as a fundamental design constraint that determines the performance ceiling and long-term reliability of various precision systems and energy conversion devices. For thermoelectric energy conversion systems, for example, stability is established as a core design criterion throughout the full device lifecycle, directly restricting long-term conversion efficiency and service durability [2]. For temperature metrology, the thermal stability of calibration baths acts as the primary traceability source, whose performance propagates to all downstream temperature measurement systems and application scenarios.

Particularly in advanced manufacturing sectors such as integrated circuit fabrication, process windows have become extremely sensitive to thermal conditions, where integrated metrology plays a pivotal role in supporting their development [3]. Consequently, developing commensurate high-precision temperature measurement and calibration capabilities is a prerequisite for promoting continuous innovation and upgrading in these industries.

The semiconductor manufacturing industry is a prime example of the demand for precise temperature control. Immersion lithography, as a key technology extending the 193 nm lithography node, enhances the numerical aperture by employing an immersion liquid, thereby achieving higher resolution [4,5]. However, the successful application of this technology critically depends on stringent environmental control, where thermal management is one of the core challenges for maintaining imaging accuracy [6]. Research indicates that minute temperature variations in the immersion liquid and optical system directly affect imaging quality, necessitating sophisticated environmental temperature control systems within lithography tools to maintain local thermal stability [7,8]. This imposes stringent requirements on the measurement reliability of numerous temperature sensors on production lines, which in turn sets near-limit performance demands on their calibration equipment: high-precision thermostatic baths.

The thermostatic bath, functioning as the core apparatus for temperature value traceability and transfer, provides a uniform and stable thermal environment. At the metrological benchmark level, Merlone et al. developed a national metrology-grade oil bath with sub-1 mK stability and uniformity in the central measurement zone, which provides a high-level traceability reference for precision temperature calibration [9]. However, such standard devices usually adopt a single heating mode without active refrigeration, and the oil medium limits their application in routine water-bath sensor calibration scenarios.

To meet the needs of frontier fields like semiconductor lithography, the performance of calibration baths must achieve the milli-kelvin (mK) level, posing significant challenges to the bath’s core subsystems [10,11]. In the field of precision manufacturing thermal management, Lu et al. proposed a circulating water cooling system based on dynamic thermal filtering, achieving ±3 mK temperature stability at room temperature [12]. Nevertheless, this split-type chiller system is designed for equipment thermal control rather than immersion sensor calibration, lacking a complete supporting temperature measurement and calibration function.

First, on the measurement front, a high-sensitivity, low-noise temperature measurement system capable of resolving micro-kelvin variations is required. Current research, such as low-noise measurement techniques based on inductive voltage dividers and AC bridges [13] and efforts in high-precision calibration and system design for negative temperature coefficient (NTC) thermistors in narrow temperature ranges [14], is dedicated to pushing the limits of measurement accuracy. Notably, while NTC thermistors are widely used for their high sensitivity [15,16], their self-heating effect is a critical factor limiting ultra-high-precision measurement. Li et al. [17] quantitatively analyzed the self-heating characteristics of NTC in multiple thermal environments, providing a theoretical basis for self-heating compensation; building upon this, domestic research teams have achieved sub-mK precision temperature measurement based on NTC by optimizing circuit designs and excitation strategies [18].

Second, on the control front, advanced control strategies capable of rapidly and accurately compensating for various thermal disturbances are needed. Advanced algorithms, such as those combining generalized predictive control with neural network PI control, have been applied in temperature control for complex equipment like lithography tools [8]. Moreover, fuzzy PID and its optimized variants [19] have demonstrated superior performance in nonlinear, time-varying systems. Additionally, intelligent methods such as fractional-order modeling and particle swarm optimization have shown promising potential for modeling and controlling water-bath systems [20], offering further avenues for intelligent control of high-precision thermostatic baths.

Finally, on the structural design front, the flow field distribution inside the bath directly determines temperature uniformity. Computational fluid dynamics (CFD) simulations combined with surrogate model optimization methods, such as the Kriging interpolation-based temperature field optimization strategy [21], have become important tools for designing high-performance thermostatic baths.

Although relevant research has been conducted and progress made domestically—for instance, in the design of multi-channel thermostatic bath systems [10], multi-sensor rapid calibration bath structure optimization [22], and advanced temperature control algorithms for lithography [8]—a gap remains in commercial mK-level high-precision thermostatic bath products compared to top-tier international standards. Currently, commercial manufacturers capable of consistently supplying ultra-stable baths that meet semiconductor-grade calibration requirements (e.g., Fluke, Hart Scientific) are predominantly foreign companies, whose products establish high technical barriers in terms of long-term stability and uniformity [9,11]. This dependence on foreign sources for such high-performance calibration equipment not only incurs high costs but also poses potential risks to supply chain security and technological autonomy.

This study addresses the critical technical gap in domestic mK-level water-bath thermostatic baths for high-precision sensor calibration. Different from single-heating metrology-grade oil baths and split industrial chillers, this work develops an integrated cabinet-type thermostatic bath with active refrigeration and heating. A high-precision NTC measurement unit based on the four-wire resistance ratio method and an optimized sub-cooling PID control logic are adopted as the core technical means to achieve full-range mK-level stability and uniformity, providing a feasible domestic solution for on-site calibration of semiconductor-grade temperature sensors.

The remainder of this paper is organized as follows. Section 2 details the overall system design, including the hardware architecture, measurement unit, and control strategy. Section 3 presents the experimental results for the prototype and analyzes key performance indicators, such as temperature stability and uniformity. Finally, Section 4 concludes the paper and suggests directions for future work.

## 2. Materials and Methods

### 2.1. Composition of the Thermostatic Bath System

**Figure 1:**
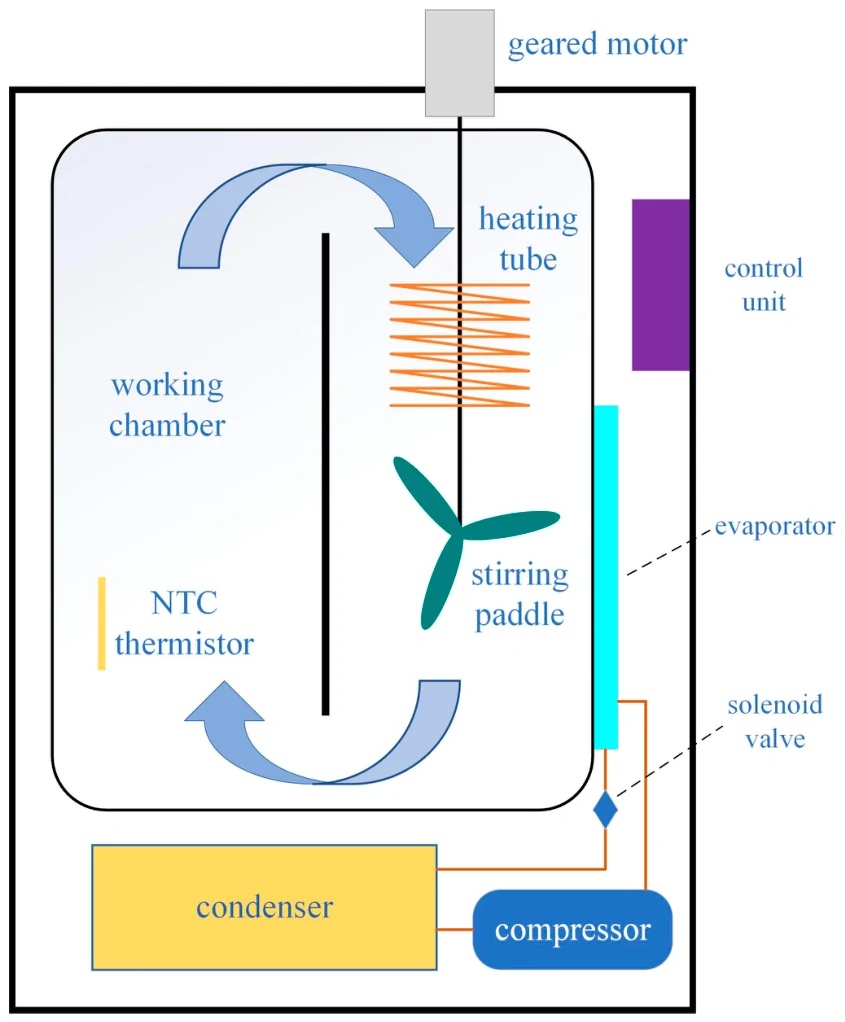
Schematic diagram of thermostatic bath system.

The principle of the thermostatic bath system is shown in Figure 1, mainly comprising a tank body, heating and refrigeration units, a stirrer, and temperature acquisition and control units. The inner tank is made of stainless steel, with an additional polyurethane insulation layer on the outside to reduce heat loss to the environment. The heater uses a spiral copper electric heating tube to ensure uniform heat distribution. The refrigeration unit adopts compressor-based cooling, exchanging heat through evaporator coils in contact with the outer wall of the inner tank to cool the water bath, and adjusts the refrigerant flow rate via a solenoid valve to change the cooling rate. The stirrer is driven by a geared motor to rotate the stirring paddle, causing liquid circulation to promote heat exchange; it operates continuously during temperature control to ensure uniform temperature distribution within the tank. The temperature sensor uses an NTC thermistor, where the resistance value is converted into a voltage by the signal acquisition circuit, and the current temperature is obtained through calculation.

The on-board controller realizes full closed-loop temperature regulation: it compares real-time collected temperature readings with preset target values and dynamically adjusts the output power of heating and refrigeration actuators. The central processing unit controls all executive components through relay drive circuits. A serial-port-connected LCD module is equipped for human–machine interaction, supporting parameter configuration, real-time operating status visualization and data display.

### 2.2. Design of the Temperature Measurement Unit

**Figure 2:**
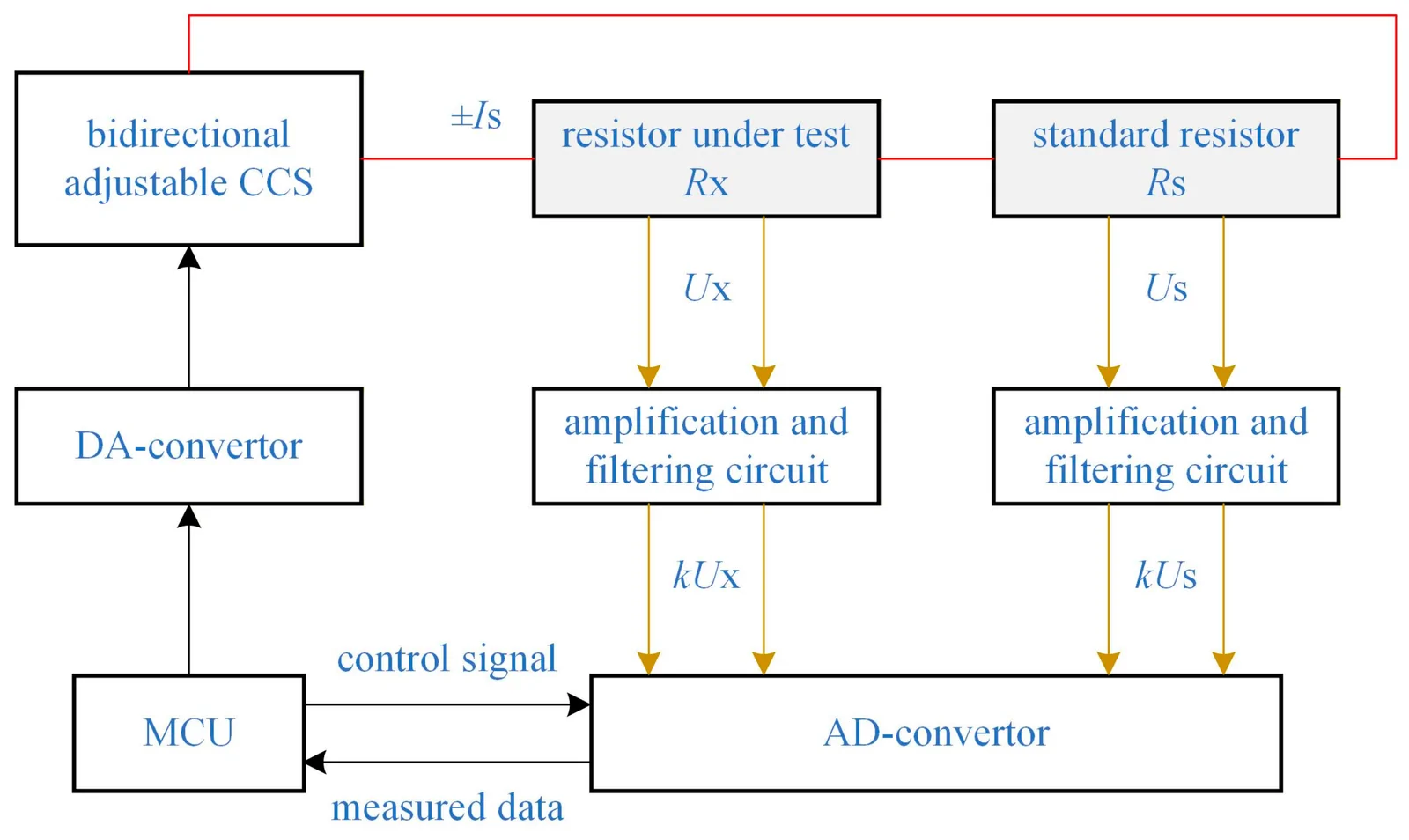
Schematic diagram of the temperature signal acquisition circuit.

Temperature measurement is a key module in the temperature control system of the thermostatic bath. The basic principle of the temperature signal acquisition circuit in this system is shown in Figure 2. It is designed using a bidirectional adjustable constant current source (CCS) with high stability and strong load capacity as the excitation source. The measured resistor *R*_X_ is connected in series with a standard resistor *R*_S_ to eliminate voltage measurement errors caused by current drift. A four-wire method is adopted, where two wires are connected at each end of the resistor for current excitation and voltage measurement respectively, eliminating measurement errors due to lead resistance and contact resistance in the circuit. Two identical signal conditioning modules are used, each channel having independent signal amplification and filtering circuits to eliminate noise effects. Additionally, a high-precision analog-to-digital converter is equipped to ensure signal acquisition accuracy.

The temperature measurement unit employs a high-sensitivity NTC thermistor with a nominal resistance of 5000 Ω (at 25 °C). Its temperature–resistance characteristic satisfies the Steinhart–Hart equation as shown in Formula (1).

(1) 1T=A0+A1In(Rt)+A 2[InRt]2+A 3[InRt]3

Here, T is the absolute temperature of the thermistor (unit: K); $R_{t}$ is the resistance of the thermistor at T (unit: Ω); $A_{0}$ to $A_{3}$ are sensor coefficients. After calibration, the sensor coefficients of the thermistor can be obtained.

**Figure 3:**
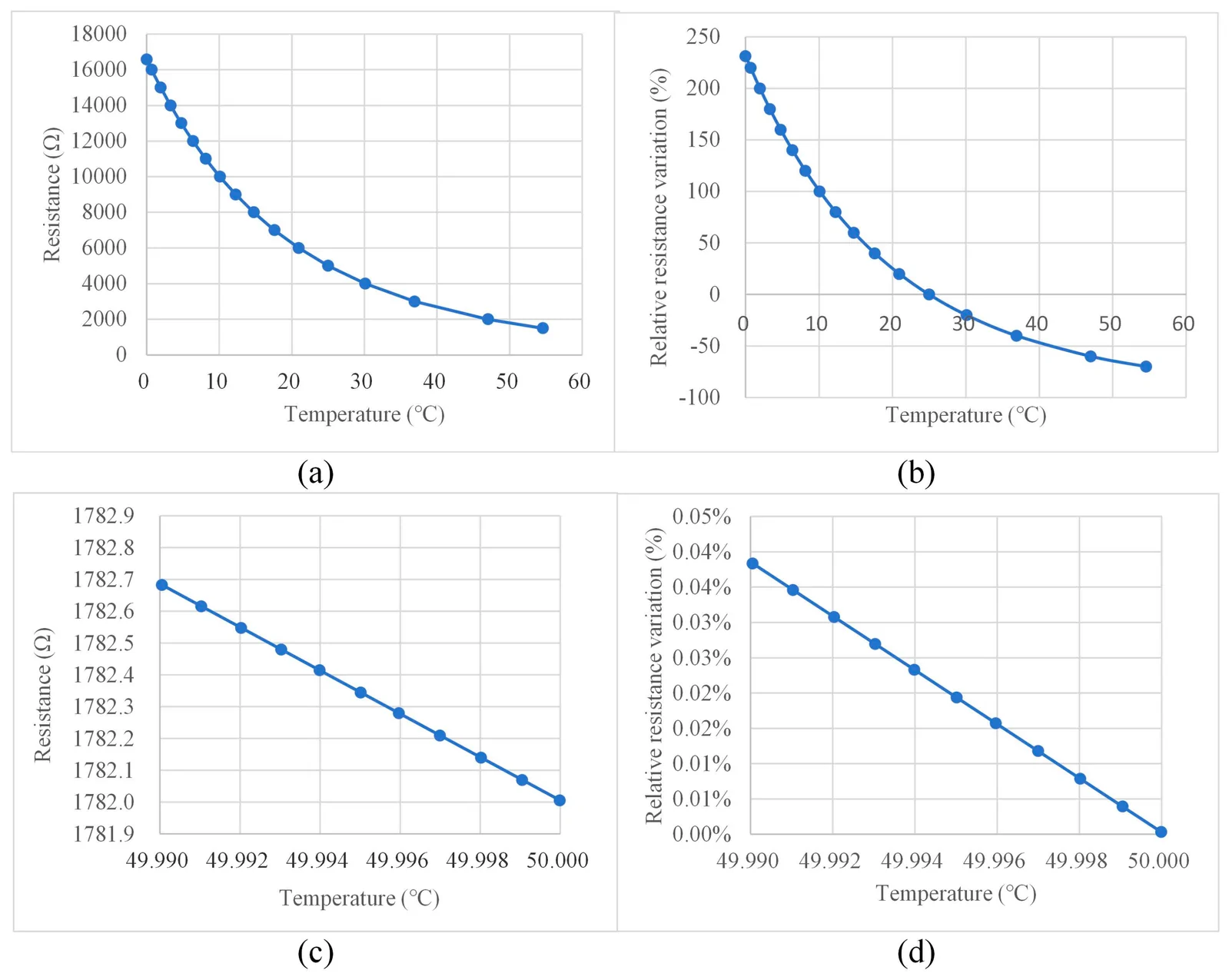
Temperature-resistance characteristics of the NTC thermistor. (**a**) Absolute resistance curve over the full operating range; (**b**) Relative resistance variation over the full range, normalized to the resistance at 25 °C; (**c**) Absolute resistance curve in the high-resolution interval near 50 °C; (**d**) Relative resistance variation in the narrow interval, normalized to the resistance at 50.000 °C.

The temperature–resistance characteristics of the calibrated NTC thermistor are presented in Figure 3, covering both the full operating range and the high-resolution narrow interval near 50 °C.

As shown in Figure 3a, the absolute resistance decreases nonlinearly with rising temperature within 0–55 °C, exhibiting a typical negative temperature coefficient feature. To eliminate the influence of the baseline resistance value and intuitively compare the temperature sensitivity in different temperature intervals, the relative resistance variation is introduced, which is defined as:

(2) $$\frac{∆R}{R_{ref}}=\frac{R\left(T\right)-R_{ref}}{R_{ref}}$$

where *R*_ref_ denotes the resistance at the specified reference temperature. For full-range characterization, the resistance at 25 °C is selected as the reference (Figure 3b), which is consistent with the nominal specification of commercial NTC devices.

To further evaluate the measurement resolution at the high-temperature end, the resistance-temperature characteristic near 50 °C is locally amplified, as shown in Figure 3c,d. Within the narrow interval of 49.990–50.000 °C, the resistance maintains a good linear negative correlation with temperature. The normalized relative variation curve (referenced to 50.000 °C) quantifies the subtle resistance change corresponding to mK-level temperature fluctuations, verifying that the designed acquisition circuit can effectively resolve such weak signals.

To reduce measurement errors caused by self-heating effects, a low excitation current should be used for the thermistor. Given that the thermistor in this system operates in a water-bath environment with good heat dissipation conditions, an excitation current of 50 µA is configured. Since the built-in constant current source of the analog-to-digital converter has weak load capacity and its current value is easily affected by load changes, a constant current source circuit based on an operational amplifier is designed. By using the reference voltage output from a digital-to-analog converter (DAC) as the excitation, the corresponding current can be obtained by adjusting the reference voltage.

Based on the above temperature-resistance relationship, calculations show that, at 0 °C (273.15 K), the thermistor resistance is approximately 16,574.02 Ω, and the potential difference across it when excited by the current is about 0.8287 V; at 50 °C (323.15 K), the resistance is approximately 1782.01 Ω, and the potential difference is about 0.0891 V. Furthermore, according to the temperature characteristic curve, the higher the temperature, the lower the corresponding resistance change rate. As shown in Figure 3c, calculations indicate that, near 50 °C, a temperature change of 0.001 °C results in a resistance change of approximately 0.07 Ω, corresponding to a voltage change of about 3.5 µV.

We adopted the resistance ratio measurement method (shown in Figure 2). A reference resistor *R*s is connected in series with the measured resistor *R*x to ensure both resistors have exactly the same current at the same time, improving measurement consistency. At time *t*1, the constant current source outputs a forward current *I*s, and the potential differences across the measured resistor and the reference resistor are measured respectively:

(3) $$U_{X1+}=\left(1+\alpha \right)\left[I_{S}\times R_{X}+E_{\theta }\right]+∆U_{0}+{∆U}^{*}$$

(4) $$U_{S1+}=\left(1+\alpha \right)\left[I_{S}\times R_{S}+E_{\theta }\right]+∆U_{0}+{∆U}^{*}$$

Here, α is the sensitivity error of voltage measurement; $E_{\theta }$ is the thermoelectric potential generated in the loop (including contact potential, thermoelectric potential, etc.); $∆U_{0}$ is the zero-point error; $∆U^{*}$ is the voltage measurement error.

At time *t*2, the constant current source outputs a reverse current −*I*s, and the potential differences across the two resistors are measured:

(5) $$U_{X2-}=\left(1+\alpha \right)\left[-I_{S}\times R_{X}+E_{\theta }\right]+∆U_{0}+{∆U}^{*}$$

(6) $$U_{S2-}=\left(1+\alpha \right)\left[{-I}_{S}\times R_{S}+E_{\theta }\right]+∆U_{0}+{∆U}^{*}$$

Combining the above equations, the measured resistance is obtained as:

(7) $$R_{X}=R_{S}\times \frac{U_{X1+}-U_{X2-}}{U_{S1+}-U_{S2-}}$$

Compared with the traditional resistance ratio measurement method, the use of forward and reverse current excitation largely eliminates the influence of related factors in the voltage measurement process.

To achieve mK-level temperature measurement accuracy, a 24-bit Σ-Δ analog-to-digital converter is selected. The ADC reference voltage of the acquisition module is 2.5 V, and the minimum voltage resolution is 0.149 µV, which is much smaller than the voltage variation corresponding to 0.001 °C within the measurement range of the thermistor, thus ensuring normal discrimination. Meanwhile, the output voltage of the thermistor falls within the effective measurement range of the ADC, guaranteeing reliable acquisition and good resolution of the temperature signal.

### 2.3. Temperature Regulation and Control Strategy

The medium temperature inside the thermostatic bath is regulated by turning on the heater and the refrigerator. The heater has a rated power of 1500 W and its power proportion is controlled via a PWM signal. The refrigerator uses a sealed compressor for cooling, with a power of about 300 W, the bath wall through evaporator coils and controlling the refrigerant flow via a solenoid valve. Depending on the error between the set target temperature and the actual temperature, three operating modes are available: heating, cooling, and constant-temperature maintenance. In addition, a compressor protection mechanism was designed with 45 °C as the stop threshold to prevent damage during high-temperature operation. According to the deviation between the set target temperature and the real-time temperature reading, the system operates in three modes: heating, cooling, and constant-temperature maintenance. The specific control logic of each mode is detailed as follows:

(a) For the heating process, if the water temperature does not exceed 45 °C: when the error between actual and target temperature exceeds 5 °C, the compressor is turned off and only the heater is activated to rapidly raise the water temperature; when the temperature error is less than 5 °C but greater than 0.5 °C, both the heater and the refrigerator are turned on to slow down the heating rate; when the temperature error is within 0.5 °C, the refrigerator remains on while the heater output power is reduced, adjusting the water temperature to a dynamic equilibrium state. When the water temperature exceeds 45 °C, the cooling function is turned off, and the heating power is controlled according to the temperature error.
(b) For the cooling process, if the water temperature does not exceed 45 °C: when the temperature error exceeds 5 °C, the heater is turned off, the compressor is turned on, and the solenoid valve is opened to increase cooling power; when the temperature error is between 0.5 °C and 5 °C, the solenoid valve is closed to reduce cooling power; when the temperature error is less than 0.5 °C, the heater power is reduced. When the water temperature exceeds 45 °C, both heating and cooling are turned off, and the system relies on natural heat dissipation to cool down below 45 °C.
(c) When the temperature error falls below 0.5 °C during heating or cooling, the system transitions to the constant-temperature maintenance mode. In this mode, both the heater and the refrigerator must be activated simultaneously to sustain a dynamic thermal equilibrium, compensating for minor heat dissipation to the surroundings. Under these conditions, the solenoid valve is closed to maintain low cooling power, establishing a sub-cooling condition. The heater power is then regulated via PID control. The PID parameters are determined using the Ziegler–Nichols auto-tuning method and are omitted here for brevity.

## 3. Results

**Figure 4:**
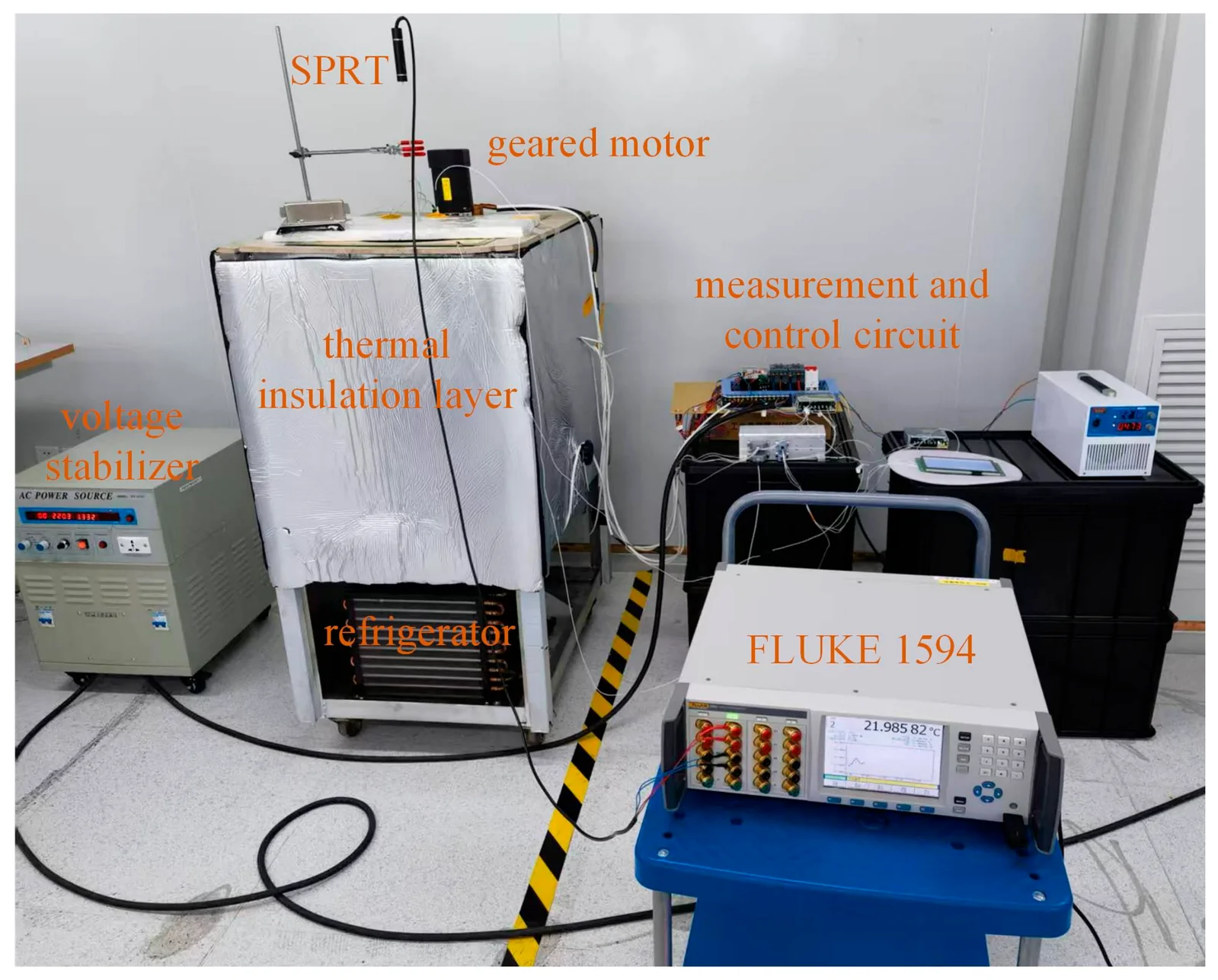
Prototype of the proposed thermostatic bath.

Based on the aforementioned design concepts, a prototype of the thermostatic bath device was constructed, as shown in Figure 4. To mitigate the impact of unstable external power supply on the temperature measurement circuit, an AC voltage stabilizer was added to power the equipment.

Experiments were conducted on the prototype to verify the thermostat bath design validity and evaluate the temperature control algorithm performance. The test procedures follow JJF 1030-2023, Measurement and Test Norm of Metrological Characteristics of Thermostatic Baths for Temperature Calibration [23], which specifies the evaluation methods for temperature uniformity, temperature fluctuation, and heating/cooling rates of liquid thermostatic baths. A standard platinum resistance thermometer (SPRT) and a FLUKE 1594 temperature measuring bridge were used to test the equipment. The thermostatic bath was evaluated in terms of temperature regulation capability, temperature stability, and temperature uniformity.

### 3.1. Temperature Regulation Test

**Figure 5:**
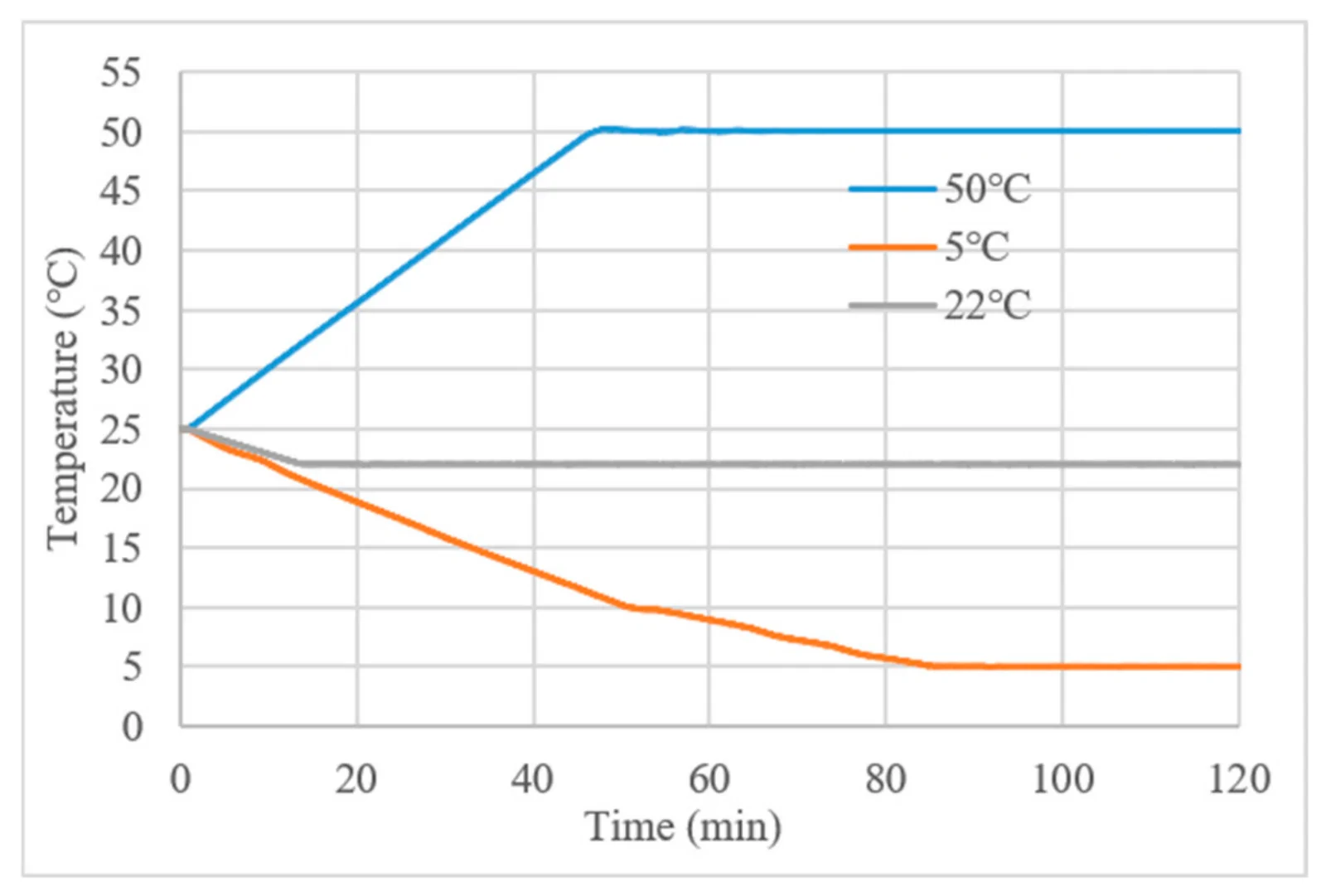
Heating and cooling temperature curves from 25 °C to 5 °C, 22 °C, and 50 °C. Sampling interval = 1 s, test duration = 120 min.

The temperature was raised or lowered from room temperature (25 °C) to three target temperatures: 5 °C, 22 °C, and 50 °C, with a sampling period of 1 s. The temperature variation curves are shown in Figure 5. When the temperature error is large during the cooling process, the cooling rate is significantly faster. Generally, it takes up to about 1.5 h (from 25 °C down to 5 °C) for the temperature field to reach a stable state.

### 3.2. Stability Test

**Figure 6:**
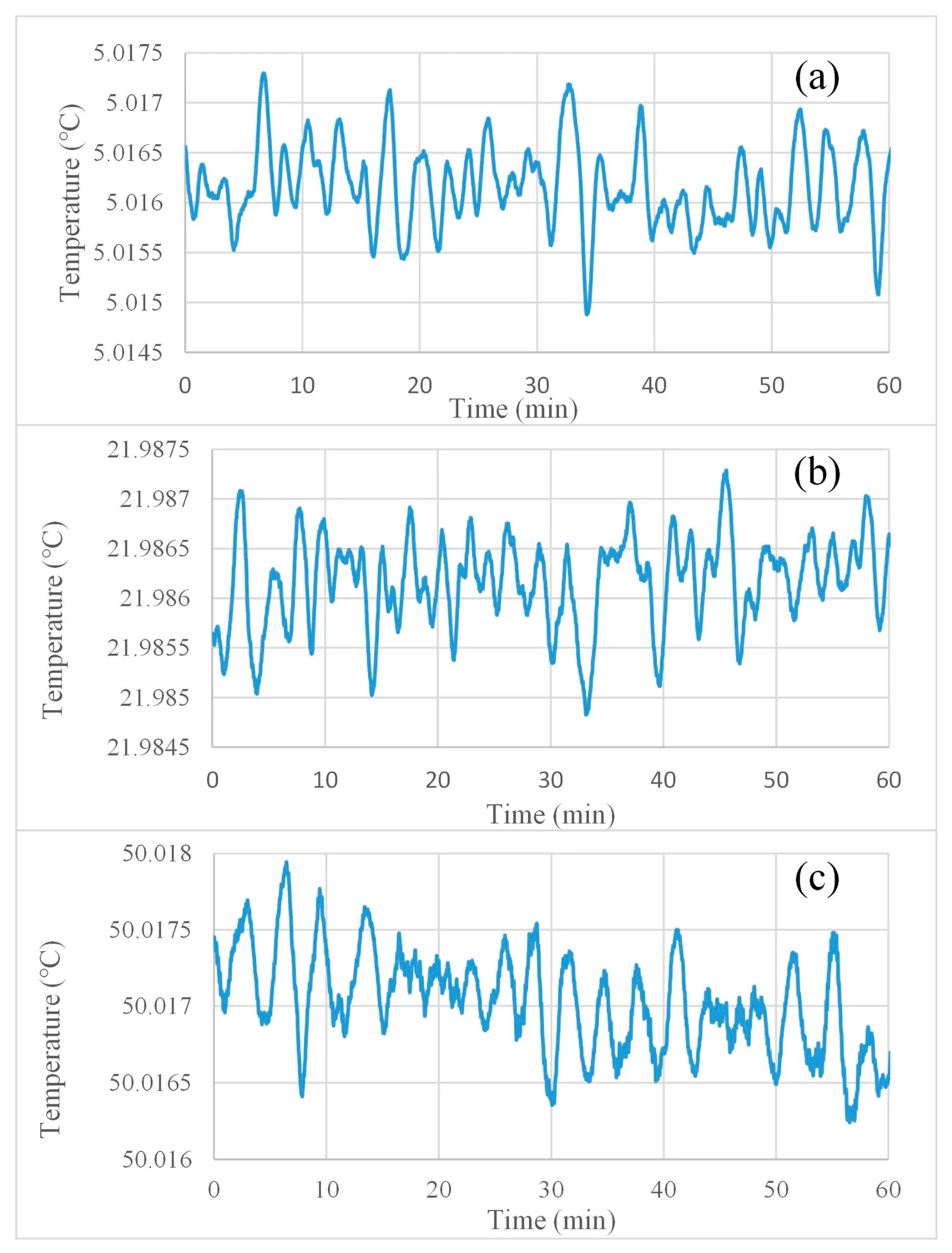
Temperature stability curves at 5 °C, 22 °C and 50 °C within 1 h. (**a**) 5 °C; (**b**) 22 °C; (**c**) 50 °C. Sampling interval: 1 s; test duration: 60 min.

The target temperature point was set and the liquid inside the bath reached a steady thermal equilibrium. A standard platinum resistance thermometer (SPRT) was placed at the geometric center of the working chamber for continuous one-hour data logging. Three representative setpoints were selected for primary stability characterization: 5 °C (lower bound of the operating range), 22 °C (typical industrial working temperature for semiconductor calibration), and 50 °C (upper bound of the operating range). The time-domain temperature fluctuation curves recorded at these three key points are presented in Figure 6.

To evaluate the repeatability of the thermostatic bath’s temperature stability performance, three fully independent one-hour steady-state tests were conducted separately at 5 °C, 22 °C and 50 °C. For each independent trial, the peak-to-peak temperature fluctuation within the full 60 min measurement window was extracted as the core stability metric. Table 1 summarizes the peak-to-peak fluctuation amplitude obtained from each repeated test, which demonstrates that the prototype maintains consistent milli-kelvin level stability over multiple independent operation cycles.

To further verify the full-range robustness of the milli-kelvin temperature control system, one-hour stability tests were supplemented at seven intermediate operating temperatures: 10, 15, 20, 25, 30, 35 and 45 °C. The maximum hourly temperature fluctuation of all test points is summarized in Table 2, demonstrating that the device maintains stability better than 3 mK across the entire 5–50 °C working range.

### 3.3. Uniformity Test

**Figure 7:**
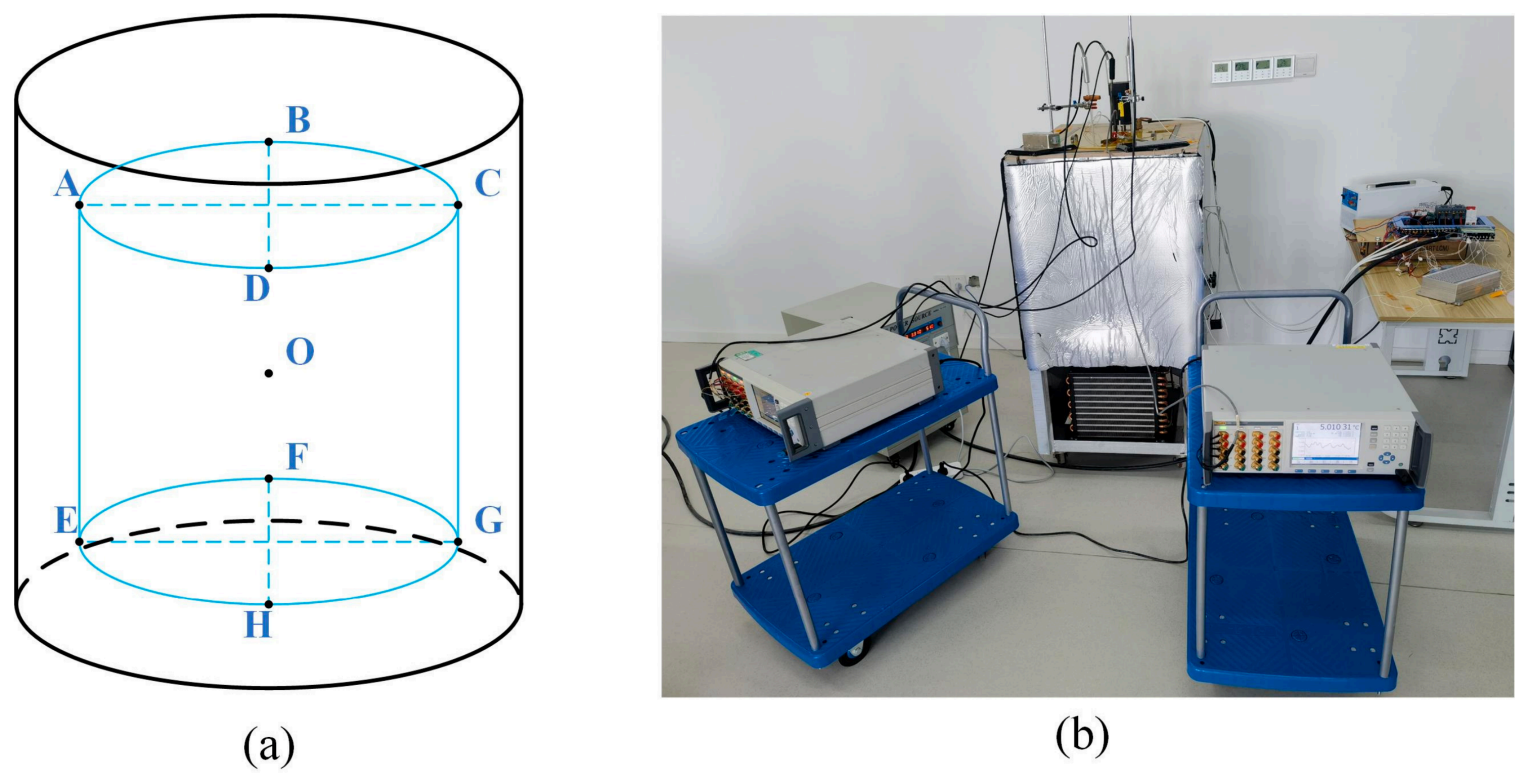
Uniformity test of the thermostatic bath. (**a**) Layout of temperature sampling points for uniformity testing. Upper/lower measurement planes are vertically separated by 200 mm (100 mm above/below center O); peripheral points share equal radial distance from O. One SPRT is fixed at O, with two movable SPRTs deployed at paired peripheral points for sequential polling measurement. (**b**) Photograph of the multi-SPRT experimental test setup.

Referring to relevant standards, the distribution of temperature sampling points inside the thermostatic bath is shown in Figure 7a. The geometric center point of the bath space is marked as O. Two planes located 100 mm from the center point serve as the upper and lower surfaces of the effective working area. On the upper surface, four points are taken at the diagonals: A, B, C, D. The four corresponding points on the lower surface are: E, F, G, H.

Three standard platinum resistance thermometers were used for testing (to reduce the influence of sensor differences on measurement results, all SPRTs were precalibrated using a water triple-point cell before the experiment). One SPRT was rigidly fixed at central point O as the reference probe for the entire uniformity test, while the other two SPRTs were sequentially placed at pairs of peripheral sampling points (e.g., points A and C in the first measurement group). Following the standard performance test specification for liquid thermostatic baths, the temperature field uniformity was quantified via reference-to-periphery temperature differences.

The FLUKE 1594 resistance bridge adopted in this work only supports sequential polling sampling with a 1 s recording interval per channel and cannot achieve truly synchronous multi-point temperature acquisition. To eliminate temperature measurement bias induced by temporal thermal drift during polling, readings were cyclically collected in the order O–A–C for four complete rounds. After averaging the four groups of recorded data for each point, two steady temperature differences, Δ_O-A_ and Δ_O-C_, were calculated to approximate the spatial deviation under an identical instantaneous thermal state.

After completing measurements for the A–C group, the two movable SPRTs were manually relocated to the next sampling pairs (B and D, E and G, F and H), and the same four-cycle polling averaging procedure was repeated for each group. This process yielded another six temperature difference values: Δ_O-B_, Δ_O-D_, Δ_O-E_, Δ_O-G_, Δ_O-F_, and Δ_O-H_. Finally, the maximum absolute value among the eight sets of differences was defined as the maximum spatial temperature difference of the thermostatic bath’s effective working zone. The multi-SPRT test setup is illustrated in Figure 7b. The uniformity test results are summarized in Table 3, where the maximum temperature deviation at all tested temperature points does not exceed 3 mK.

Beyond the temperature metrological performance characterized above, key physical parameters of the prototype are provided for engineering reference. The bath has a working chamber of Φ300 mm × 350 mm with an internal volume of 34 L. Its overall outer dimensions are 858 mm × 796 mm × 842 mm with a net weight of approximately 160 kg. The total rated power is 2.5 kW under 220 V/50 Hz mains supply.

### 3.4. Uncertainty Analysis of the Calibration System

In this section, a comprehensive uncertainty evaluation of the calibration system is carried out according to JCGM 100:2008 [24] to quantitatively verify the mK-level calibration performance.

Four independent uncorrelated error sources are identified for the whole comparison calibration system, including the traceable standard SPRT, the measuring bridge, bath temporal temperature fluctuation and bath spatial temperature difference. Type-B evaluation with rectangular distribution is adopted for all components as described below.

(1) The Class 1 SPRT acts as the core traceability standard of the calibration chain and contributes the primary reference uncertainty. Its short-term temperature drift is guaranteed to be less than 0.5 mK, which is treated as a rectangular distributed limit interval. The corresponding standard uncertainty is calculated as:

(8) $$u_{SPRT}=\frac{0.5}{\sqrt{3}}\approx 0.289\;mK$$

(2) The FLUKE 1594A super thermometer serves as the resistance reading device. Its specified maximum permissible error is ±0.5 × 10^−7^ °C. Converted via rectangular distribution, the standard uncertainty of the bridge is approximately 2.89 × 10^−5^ mK, which is four orders of magnitude smaller than other uncertainty components. Its contribution is negligible and excluded from subsequent combination calculation.
(3) Temporal temperature fluctuation of the thermostatic bath introduces extra uncertainty during continuous sensor calibration. A conservative peak-to-peak fluctuation limit of △*T*_stab_ = 3 mK is adopted to cover the full temperature variation range within a 1-h measurement cycle. Assuming rectangular distribution over the fluctuation range, the standard uncertainty induced by stability is expressed as:

(9) $$u_{stab}=\frac{∆T_{stab}}{\sqrt{12}}\approx 0.866\;mK$$

(4) Spatial temperature nonuniformity across the effective working zone brings another environmental uncertainty source. The reference SPRT and device under test cannot be placed at identical spatial coordinates, and a conservative maximum spatial temperature difference △*T*_uni_ = 3 mK is used for estimation. This temperature offset follows rectangular distribution, and its standard uncertainty is calculated via:

(10) $$u_{uni}=\frac{∆T_{uni}}{\sqrt{12}}\;\approx \;0.866\;mK$$

The combination formula for uncorrelated uncertainty components is written as:

(11) $$u_{c}=\sqrt{u_{SPRT}^{2}+u_{stab}^{2}+u_{uni}^{2}}$$

As shown in Table 4, the expanded uncertainty is calculated as *U* = 2*u*_c_ ≈ 2.52. The same conservative limit values are applied for calculation at other points, and the system expanded uncertainty remains below 3 mK at all working points.

Even under the conservative upper limits of bath fluctuation and spatial temperature difference, the total uncertainty of the complete calibration system is controlled within 3 mK, enabling high-precision comparison calibration of temperature sensors at the milli-kelvin level.

### 3.5. Quantitative Performance Comparison with State-of-the-Art Devices

To objectively evaluate the performance level and technical advantages of the proposed calibration system, three representative thermostatic devices reported in recent years are selected for quantitative comparison, covering different precision grades and application scenarios. All parameters are directly extracted from the original literature to ensure authenticity and rigor. The comparison results are summarized in Table 5.

As can be seen from the comparison, the metrology-grade oil bath [9] achieves the highest accuracy, with sub-1 mK stability over 12 h. Nevertheless, it serves as a national temperature traceability standard with a single heating mode and oil medium, which is not suitable for daily low-temperature water-bath sensor calibration. The circulating water cooling system [12] reaches mK-level stability at room temperature, but it adopts a split structure without an integrated calibration acquisition function. The industrial multi-sensor bath [22] focuses on portable calibration efficiency, yet its stability is an order of magnitude lower than that of this work, making it difficult to meet the calibration requirements of high-precision sensors. Compared with those devices, the proposed system realizes integrated refrigeration and heating in a single cabinet and maintains mK-level stability and uniformity throughout the full (5–50) °C working range. This integrated design breaks the scenario limitations of existing high-precision platforms: it retains the accuracy level close to metrology-grade reference baths while supporting convenient on-site water-bath calibration. In terms of domestic equipment, its accuracy is nearly two orders of magnitude higher than conventional industrial calibration baths, filling the performance gap of domestic mK-level temperature calibration equipment for semiconductor applications. Notably, the current 5–50 °C operating range is tailored to the core demand of room-temperature calibration of semiconductor-grade temperature sensors, and expanding the temperature range is a key direction for subsequent product iteration.

Regarding application prospects, emerging high-sensitivity temperature sensing devices represented by carbon-based composite thermistors and two-dimensional material-based dual-mode sensors have attracted extensive research attention in recent years [25,26]. Polymer–carbon composite temperature sensors with ultra-high linearity usually have a high temperature coefficient of resistance and sub-mK resolution potential, and their sensitivity calibration highly relies on an ultra-low-fluctuation temperature baseline. For InSe-based dual-mode sensors integrating gas and temperature sensing functions, the characterization of cross-sensitivity between different modes also imposes strict requirements on the spatial uniformity of the calibration temperature field. The mK-level stable temperature field and spatially uniform working chamber provided by the proposed system can serve as a reliable temperature reference for static sensitivity calibration, temperature drift characterization and multi-sample consistency verification of such novel sensors, effectively supporting the performance research and batch calibration of new-generation integrated sensing systems.

## 4. Conclusions

Addressing the calibration requirements of temperature sensors in high-precision thermometry, this study proposes a thermostatic bath system with mK-level temperature control performance. Optimizations were carried out in aspects such as system design, core temperature measurement module, and temperature control logic, and a prototype device was subsequently constructed. Experimental results show that the designed thermostatic bath has an effective temperature control range of (5–50) °C, a stability of less than 3 mK per hour, and a uniformity better than 3 mK. Compared with existing high-precision calibration platforms, this system features an integrated refrigeration–heating structure and full-range mK-level performance and can support on-site calibration of semiconductor-grade temperature sensors.

Future work will focus on two directions. First, multi-physics simulations will be adopted to optimize the internal flow and temperature fields for better thermal performance. Second, schemes for expanding the operating temperature range will be investigated: for the low-temperature end, antifreeze medium with cascade refrigeration will be adopted to extend the lower limit; for the high-temperature end, in addition to high-temperature heat transfer oil and evaporation suppression structure, the temperature measurement unit will be optimized to address the reduced sensitivity of NTC thermistors at high temperatures, so as to cover wider calibration scenarios.

## Figures and Tables

**Table 1:** Repeatability of 1-h stability peak-to-peak fluctuation at three representative temperature points.

Setpoints (°C)	1st Test (mK)	2nd Test (mK)	3rd Test (mK)
5	1.8	1.8	2.4
22	2.3	2.1	1.7
50	1.3	1.2	1.7

**Table 2:** One-hour temperature stability at tested setpoints across the full operating range.

Setpoint Temperature (°C)	Maximum Temperature Fluctuation (mK)
5	2.4
10	2.3
15	2.6
20	2.5
25	2.5
30	2.9
35	1.7
40	2.0
45	1.3
50	1.7

**Table 3:** Test results of temperature uniformity in the thermostatic bath (mK).

Temp.	Δ_O-A_	Δ_O-C_	Δ_O-B_	Δ_O-D_	Δ_O-E_	Δ_O-G_	Δ_O-F_	Δ_O-H_	Max. Δ
5 °C	2.2	0.9	1.3	1.1	0.2	1.4	0.3	0.1	2.2
22 °C	0.5	0.1	1.3	1.9	0.1	0.7	1.4	0.5	1.9
50 °C	0.3	1.3	0.5	0.1	0.3	0.8	0.2	1.5	1.5

**Table 4:** Uncertainty budget of the calibration system at 22 °C (mK).

Uncertainty Component	Symbol	Standard Uncertainty
Calibrated Class 1 SPRT	*u*_SPRT_	0.289
FLUKE 1594A	*u*_bridge_	2.89 × 10^−5^
Bath temporal stability	*u*_stab_	0.866
Bath spatial uniformity	*u*_uni_	0.866
Combined standard uncertainty	*u*_c_	1.258
Expanded uncertainty (*k* = 2)	*U*	2.52

**Table 5:** Performance comparison of typical thermostatic calibration devices.

Reference	Working Temperature Range	Peak-to-Peak Stability	Spatial Uniformity	Core Features and Limitations
Merlone et al. [9]	(−10~100) °C	<1 mK (12 h)	<1 mK (central 10 cm zone)	Metrology-grade primary standard oil bath; single heating only, no active refrigeration.
Lu et al. [12]	Only verified at 22 °C	±3 mK (@ 22 °C, 1 h)	Not reported	Split circulating water chiller; for precision manufacturing thermal management; no integrated sensor calibration function.
Jiang et al. [22]	(0~100) °C	≤200 mK (10 min)	4 mK (@ 50 °C)	Portable industrial multi-sensor calibration bath; low accuracy grade.
This work	(5~50) °C	<3 mK (full range, 1 h)	<3 mK (effective working zone)	Integrated compressor refrigeration and heating; dedicated for precision resistance sensor calibration

## Data Availability

Data are not publicly available and can be obtained by contacting the corresponding author if necessary.
